# Measuring the Marginal Gap of Pre-Cemented All-Metal Single Crowns: A Systematic Review of In Vitro Studies

**DOI:** 10.3390/dj13050204

**Published:** 2025-05-02

**Authors:** James Dudley, Taseef Hasan Farook

**Affiliations:** Department of Prosthodontics, Adelaide Dental School, The University of Adelaide, Adelaide, SA 5005, Australia

**Keywords:** crown marginal gap, fit discrepancy, all-metal crown, measurement techniques, systematic review

## Abstract

**Background:** Different methods have been used to fabricate and measure marginal gap in all-metal crowns, yet a systematic review on this topic has not been conducted. **Objective:** To review the existing literature regarding the measurement methods employed for the in vitro marginal gap measurement of pre-cemented all-metal single crowns and examine the influence of crown fabrication method on the marginal gap. **Materials and Methods:** A systematic search was performed from December 2024 backwards across EBSCO Host, Scopus, PubMed, and Web of Science databases following the Preferred Reporting Items for Systematic Reviews and Meta-Analyses (PRISMA) guidelines and predefined eligibility criteria. The quality of included articles was evaluated using the Joanna Briggs Critical Appraisal Checklist. **Results:** Ten studies, involving 180 crowns, assessed marginal gaps using computerised superimposition (102 µm), scanning electron microscopy (89 µm), profilometry (100 µm), photogrammetry (59 µm), impression replica techniques (124 µm), and direct view microscopy (35 µm). Marginal gaps varied across crowns constructed with cobalt–chromium (97 µm), titanium (56 µm), noble metals (127 µm), and base metal alloys (35 µm). No significant differences (t = 1.06, *p* = 0.315) were observed between CAD/CAM (103.21 ± 58.56 µm) and lost wax casting method (71.59 ± 43.94 µm) of crown fabrication when analysed using an independent *t*-test. **Conclusions:** Cobalt–chromium was the most used material for AMCs, while titanium alloys produced the lowest mean marginal gap per crown. No significant differences in reported marginal gaps were observed between crowns fabricated using lost wax casting and CAD/CAM techniques. However, the limited number of studies, variation in measurement methods, and inconsistency in methodological rigour restricted the generalisability of the findings.

## 1. Introduction

All-metal crowns (AMCs) provide benefits of strength, durability, and resistance to chipping and fracture. In the aesthetically focussed modern era of advanced technologies and increased use of tooth coloured crown materials, AMCs remain a restorative option often recommended on account of the conservative reduction required in the tooth preparation process [1,2]. While their popularity may be declining, AMCs still provide an important option in restorative dentistry.

The traditional lost wax technique for constructing AMCs is labour-intensive and technique-sensitive [1]. More recently, computer-aided design and computer-aided manufacturing (CAD-CAM) technology have been employed to fabricate AMCs, delivering results comparable to those achieved with traditional methods [2]. Additionally, metal 3D printing has been applied to produce cobalt–chromium alloy AMCs with marginal gap accuracy comparable to AMCs constructed using conventional casting techniques, therefore offering a reliable alternative [3].

The marginal gap is the space between the axial wall of the prepared tooth and the internal surface of the restoration at the margin [4]. The marginal fit describes how precisely the crown conforms to the edge of the tooth. An ideal crown should exhibit a minimal and uniform marginal gap, preventing the accumulation of bacteria or debris, which in turn enhances the restoration’s durability and clinical success. Among aesthetics and fracture resistance, the marginal fit of a restoration is considered the most important factor for the success of crowns [5,6,7]. Precise marginal fit reduces the risk of plaque accumulation and hypersensitivity from microleakage, while suboptimal marginal fit can predispose to periodontal disease, caries, pulpitis, and increased cement exposure to the oral environment [6,7,8]. The factors that influence the marginal fit of a restoration include preparation design, finish line location, restorative material, fabrication method, impression material, and impression technique [9,10,11,12,13,14,15].

Measuring the marginal gap of pre-cemented AMCs is critical to assessing fit and overall quality as the precision of marginal fit directly impacts the longevity and clinical success. The accuracy of the marginal fit significantly influences the durability and clinical success of restorations [16]. Accurate measurement of the marginal gap assists clinicians and dental technicians to evaluate the fabrication process and make necessary modifications.

The numerical value for an acceptable marginal gap has changed over time and differs depending on the type of crown. Traditionally, clinical marginal gaps of up to 120 µm [17] have been considered acceptable, though it was acknowledged that margins greater than 160 µm could potentially be clinically successful [18]. This work was based on in vitro measurements using polyether rubber films of in vivo seated restorations [17]. However, recent research has suggested more stringent acceptable gaps such as ≤100 µmm [19,20], ≤90 µm [21], and ≤75 µm [22]; however, different materials, fabrication methods, and measurement methods have been used [23,24,25]. Despite these findings, there is no consensus on the ideal marginal gap [26].

Marginal gap measurements are usually made at convenient locations and the measurements averaged to infer the overall crown marginal gap [4,27,28]. It has been recommended to make at least 50 measurements per crown to reduce potential errors; however, this was based on calculations of the arithmetic mean and linked to the variation in gaps and desired precision in a small sample size [29]. There is currently no consensus on the ideal number of measurements needed for an accurate evaluation of crown marginal gap [30,31,32].

Methods for measuring marginal gaps can be categorised as two-dimensional (2D) or three-dimensional (3D), and destructive (DE) or non-destructive (ND), and the main measurement methods include direct view microscopy (2D, ND), scanning electron microscopy (3D, ND), and impression replica (2D, ND). There have been conflicting reports comparing the accuracy of scanning electron microscopy with light microscopy in measuring crown marginal gap [33,34]. Despite numerous reviews investigating crown marginal gap assessment and its influencing factors, a thorough comparison of the different measurement methods has not been conducted [15,22,24,26,35,36,37].

The aim of this study was to systematically review the existing literature regarding the measurement methods employed for in vitro marginal gap measurement of pre-cemented all-metal single crowns. Moreover, this study aimed to determine whether variables such as the crown fabrication method influenced the measured marginal gap. It was hypothesised that there would be no significant difference in marginal gaps between crowns fabricated using the traditional lost wax technique and those produced via CAD/CAM.

## 2. Materials and Methods

This systematic review was performed in accordance with the Preferred Reporting Items for Systematic Reviews and Meta-Analyses (PRISMA). The study protocol was registered with Open Science Framework (https://osf.io/y5xah; accessed on 9 April 2025). The inclusion criteria concentrated on in vitro studies that examined marginal gaps of complete-coverage single AMCs pre-cementation. Eligible studies were required to provide a detailed methodology for gap assessment, including the number of measurements performed. Crown types considered included laboratory-fabricated crowns made of any metal or metal alloy, such as noble metals, base metal alloys, gold alloys, stainless steel, or titanium. Only studies that measured pre-cementation marginal gaps using a consistent method throughout the experiment were included to ensure comparability of results.

The exclusion criteria ruled out in vivo studies involving complete coverage all-ceramic crowns, metal–ceramic crowns, or crowns constructed by layering secondary materials onto cast alloys, as well as crowns evaluated virtually without a physical component. Studies assessing marginal gaps on preformed metal crowns, paediatric crowns, partial crowns, fixed partial dentures, or endocrowns were also excluded. Additionally, studies relying solely on manual visual or tactile estimations of marginal gaps or those focusing solely on internal fit without addressing marginal gaps were deemed ineligible.

Data were collected from the following databases: Scopus^®^, PubMed.gov, EBSCOHost Dentistry and Oral Sciences Source (DOSS), and Web of Science™ (WoS). Web of Science™ includes multiple indexed databases, such as the Web of Science Core Collection™, Current Contents Connect^®^, Derwent Innovations Index™, KCI-Korean Journal Database™, MEDLINE^®^, Russian Science Citation Index™, and SciELO Citation Index™, with translations into English where applicable. Searches were performed from December 2024 backwards, with no restrictions on publication dates. Field-specific filters were applied in Scopus and Web of Knowledge to focus on research related to material sciences in dentistry and healthcare.

The search process was independently conducted by two reviewers (JD and THF), and inter-rater reliability was assessed using the Cohen’s kappa (K) coefficient. The methodology incorporated keyword-based logic grids, Boolean operators, and manual reviews of bibliographies from related publications. Below is an example search query used for EBSCOHost DOSS.

(TI(Fit OR Gap* OR Space OR Distance OR Length* OR Accuracy* OR Precision) OR AB(Fit OR Gap* OR Space OR Distance OR Length* OR Accuracy* OR Precision)) AND (TI(“internal margin*” OR “internal discrepancy*” OR “margin* adaptation*” OR “cervical margin*” OR preparation OR “margin* integrity” OR “margin* opening*” OR “edge gap*” OR “margin* gap*”) OR AB(“internal margin*” OR “internal discrepancy*” OR “margin* adaptation*” OR “cervical margin*” OR preparation OR “margin* integrity” OR “margin* opening*” OR “edge gap*” OR “margin* gap*”)) AND (TI(“Un-cemented” OR “Fixed dental”) OR AB(“Un-cemented” OR “Fixed dental”) OR DE “Dental Crowns”) AND (TI(titanium* OR gold OR alloy OR noble OR metal OR “base metal” OR “Ni-Cr” OR Nickel OR chromium) OR AB(titanium* OR gold OR alloy OR noble OR metal OR “base metal” OR “Ni-Cr” OR Nickel OR chromium)) NOT (TI(PBM OR “Porcelain fused to metal” OR “Porcelain bonded to zirconia” OR “PBZ” OR ceramic OR laminate* OR veneer OR inlay OR onlay OR implant OR monolithic OR Emax OR “Lithium disilicate” OR dentures) OR AB(PBM OR “Porcelain fused to metal” OR “Porcelain bonded to zirconia” OR “PBZ” OR ceramic OR laminate* OR veneer OR inlay OR onlay OR implant OR monolithic OR Emax OR “Lithium disilicate” OR dentures)).

Eligible manuscripts were screened using the Covidence systematic review platform (Covidence.org; Veritas Health Innovations Ltd., Melbourne, Australia), which facilitated automated removal of duplicates and ensured consensus among reviewers. Disagreements were resolved prior to advancing eligible articles for further review, ensuring full agreement on the final selection. To ensure comprehensive coverage, all relevant studies were included for a broad narrative summary. The quality of the included studies was assessed by a prosthodontist (JD) using the Joanna Briggs Institute Critical Appraisal Checklist for Analytical Cross-Sectional Studies. No studies were excluded based solely on quality assessment results.

The following parameters were recorded for analysis: the number of crowns fabricated, crown material, method of crown construction, underlying crown preparation material, tooth form measured, measurement methods used, the number of marginal gap measurements per crown, the reported range of marginal gaps (in µm) across any direction, and the mean marginal gap for AMCs (rounded to the nearest whole number, in µm).

Statistical analyses were performed using SPSS software (version 26; IBM Corp, Armonk, NY, USA). The Shapiro–Wilk test was used to assess normality (*p* = 0.579). An independent *t*-test was conducted to compare the overall means of the marginal gaps across fabrication methods, namely, CAD/CAM and lost wax casting. This was followed by a robust equality of means test to evaluate the influence of data distribution on group outcomes.

## 3. Results

**Figure 1:**
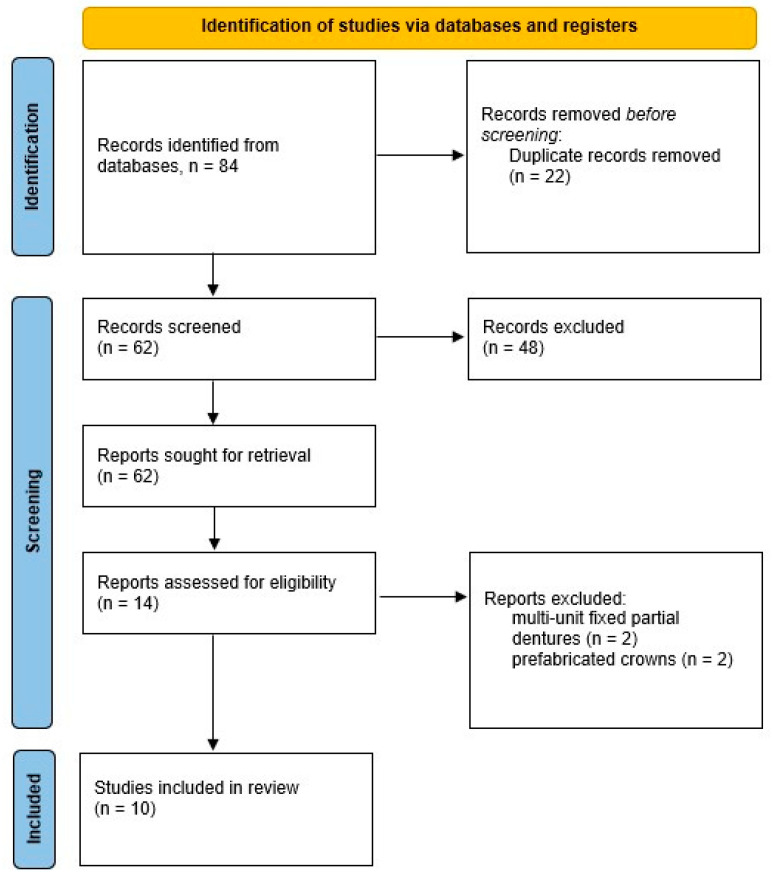
PRISMA flowchart showing search results.

Ten articles were included for appraisal, as outlined in the PRISMA flow diagram (Figure 1), involving the marginal gaps of 180 crowns. Six studies evaluated the marginal gaps of cobalt–chromium alloy crowns (97.17 ± 55.73 µm) [38,39,40,41,42,43], which was the material used in all included studies published within the past 10 years. Earlier studies (prior to 2014) documented assessments of AMCs fabricated with titanium (55.85 ± 30.57 µm) [44,45], noble metals (127 µm) [46], and base metal alloys (35 µm) [47]. Computer-aided design–computer-aided manufacturing (CAD-CAM) was the most used AMC fabrication method, followed by lost wax casting and laser sintering. The findings have been summarised in Table 1. A critical appraisal (Table 2) revealed that five of the ten articles [38,39,40,45,46] did not fully address all of the identified confounding factors that could have influenced the measurement of the marginal gap. An important consideration is the inconsistency in the underlying materials of the dies used to fabricate the crowns, which introduces a significant risk of variation and bias, thereby impacting the overall certainty of the evidence.

The most frequently used method for measuring the marginal gap was 3D superimposition, used in four studies; however, seven different measurement methods were used across the ten studies. The measurement methods used to measure marginal gaps (and their resultant mean marginal gap) included computerised superimposition (102 µm), scanning electron microscopy (89 µm), profilometry (100 µm), photogrammetry (59 µm), impression replica techniques (134 µm), and direct view microscopy (35 µm). A statistical comparison across these measurement methods was not feasible due to the limited data and the unequal distribution of measurements across the methods applied.

Assuming the accuracy of these measurement methods and materials used for crown fabrication was comparable [18], variations in the traditional lost wax technique and CAD/CAM in AMC fabrication were analysed (Table 3). Levene’s test for equality of variance was not significant (F = 0.381, *p* = 0.551).

The number of marginal gap measurement points made per crown was not reported in all studies. The most documented underlying abutment tooth used for AMC fabrication was an acrylic tooth, and the most frequently used tooth form was a maxillary molar, used in five studies.

## 4. Discussion

This systematic review found that only ten studies reported on the in vitro marginal gap measurement of pre-cemented single AMCs. Clinical significance is underpinned by in vivo research, and with the lack of in vitro studies, clinicians and researchers are directed to lower levels of evidence that may not have scientific and clinical rigour. With AMCs still holding clinical significance, the robustness of research in this area requires development and standardisation to develop reliable guidelines for clinical practice.

The various measurement methods have certain limitations that could have influenced the results [18,37]. The most frequently used method for measuring the marginal gap of pre-cemented AMCs was 3D superimposition. This method involves overlapping 3D scans and measures volume rather than points; therefore, marginal gap measurement requires 2D sections of the 3D analysis to measure points [48]. Three-dimensional superimposition offers the benefits of convenience, practicality, and repeatability but requires specialised and costly equipment.

The direct-view microscopy method is susceptible to errors due to the selection of measurement points and the differentiation between materials. It also relies on magnification and is restricted to in vitro use, which may not fully replicate clinical conditions. Additionally, the magnification process can introduce inaccuracies if the measurement points are not precisely chosen or if there is difficulty distinguishing between different materials. The impression replica method, while useful, may encounter difficulties in pinpointing the location of crown margins. This tool can also suffer from tearing of the elastomeric film, which can lead to inaccuracies. Errors in sectioning the impression can further contribute to overestimated measurements, as the process may not capture the true dimensions of the marginal gap. While profilometry is non-destructive, it necessitates accurate repositioning to avoid discrepancies. Any slight misalignment during repositioning can result in discrepancies, affecting the reliability of the measurements. The large range of different measurement methods used across the included studies indicates a lack of standardisation in method selection, likely dictated by availability and cost.

In addition to these tool-specific limitations, frequent confounding factors included the uneven distribution of samples among groups and the variability in measurement tools, each with its own limitations, as previously mentioned [18]. Moreover, inconsistencies in specimen preparation, differences in cement space, and variations in alloy composition introduced biases. The absence of standardised protocols across studies further complicated result comparisons, potentially impacting the accuracy and reliability of the findings. These confounding factors underscore the necessity for more rigorous and standardised methodologies in future research. Establishing consistent protocols for sample preparation and measurement methods across research teams could greatly improve the comparability and reliability of the results. Additionally, addressing uneven sample distribution through better study designs and larger sample sizes would lead to more robust and generalisable findings.

Seven [28,38,39,40,41,45,47] of the ten included studies reported mean marginal gaps within the generally recognised clinically acceptable limit of 120 µm [17]. The study by Mai et al. [42] seemingly represents an outlier (reporting a mean marginal gap of 200 µm) and the Ferrari et al. [46] study did not fully address all identified confounding factors. The accuracy of different measurement methods has not been compared, and a statistical analysis was not possible in this study due to the limited data, which impedes meaningful comparison across the included studies. Consequently, clinicians and researchers must rely on individual study findings to assess marginal gaps against established standards [17], clinical guidelines that suggest <80 µm is difficult to detect [22], or personal preferences [31]. Despite smaller gaps being preferable, some studies indicate that bonding materials to tooth surfaces may reduce the importance of the marginal gap [37]. The optimal marginal gap for AMCs, and indeed for crowns in general, remains uncertain.

The average number of crowns evaluated in each study was 16, which falls below the suggested 50 measurements previously recommended [29]. Without explicit justification in the included research, it appears convenience was the primary factor in determining the sample size, rather than scientific rationale. A critical aspect of further research should focus on establishing an appropriate number of samples and measurements per sample, instead of simply citing previous studies to justify methodological choices.

The most used AMC material was a cobalt–chromium alloy, which was the only material used in the five included studies published in the past 10 years. Congruently, CAD/CAM was the most used method of AMC fabrication. This represents a change to the traditional AMC fabrication philosophy that used the lost wax technique with precious or semi-precious alloys. The fabrication process using CAD/CAM milling can be either additive or subtractive, which helps to eliminate the issues of porosity and imperfections typically associated with casting techniques [11,12]. However, there are multiple CAD/CAM design and milling unit variables that can influence the marginal gap, such as cement space settings [49]. Nevertheless, within the limitations of this study, the method of fabrication did not influence the marginal gap of the AMCs, based on the assumption that the accuracy of the measurement methods was comparable. It is difficult to provide a scientific rationale for the lack of difference in the AMC marginal gap across the different fabrication methods considering the data distribution. The large range of experimental variables (materials, fabrication methods, measurement methods, and number of measurements) used in the included studies reduced the statistical analysis meaning; therefore, this observation should be observed with some caution.

The current study focused on the in vitro measurement of pre-cemented AMCs. The statistical analysis was constrained by experimental variables, crown construction techniques, and variations in measurement methods, which resulted in significant mean variations offset by large standard deviations. Although in vitro analysis provided a controlled environment for assessing marginal gaps, it could not account for factors such as preparation design and clinically used impression materials, highlighting a limitation of in vitro studies on crown marginal gaps. Evaluating the finish line location was deemed superfluous as the classification of supra- or sub-gingival margins holds little relevance in an in vitro context. This limitation highlights the need for future research to standardise the marginal gap measurement methods and protocols to ensure accuracy, regardless of the measurement methods used. Future studies should also aim to transition from in vitro to in vivo measurements to better reflect clinical conditions. Standardising these methods will help achieve more reliable and consistent results, ultimately improving the clinical success and longevity of AMCs. By addressing these limitations, researchers can enhance the understanding of marginal gaps and their impact on AMCs. This study provides a reference point for future research.

## 5. Conclusions

The most frequently used method for measuring the marginal gaps of pre-cemented all-metal crowns was 3D superimposition, used in four of the ten included studies; however, seven different measurement methods were used across the ten studies. The most prevalent all-metal crown fabrication method was CAD/CAM using a cobalt–chromium alloy, reported in all five of the included studies published in the past 10 years. Within the studies included in this systematic review, the crown fabrication method did not influence the marginal gap of all-metal crowns.

## Figures and Tables

**Table 1:** Marginal gap assessment for all-metal crowns.

Author, Year	Number of Crowns Fabricated	Crown Material (s)	Method of Crown Construction	Underlying Crown Preparation Material	Tooth Form Measured	Measurement Method	Number of Marginal Gap Measurements per Crown	Mean Range of Reported Marginal Gap Measurements in Any Direction		Overall Marginal Gap of Metal Crowns (in µm, Rounded to Nearest Whole Number)
								Assessments	Marginal Gap (in µm)	
Berger, 2022 [38]	30	Cobalt–chromium	CAD/CAM	Metal die	Maxillary molar	2D and 3D superimposition	-	2D superimposition	34 to 45	49
								3Dsuperimposition	28 to 90	
Chou, 2021 [43]	30	Cobalt–chromium	CAD/CAM CNC milling Lost wax casting	Acrylic tooth model	Mandibular molar	Impression replica	2	CAD/CAM	111.3 ± 12.3	75
								CNC milling	93.79 ± 10.47	
								Lost wax casting	121.18 ± 16.25	
Gholamrezaei, 2020 [39]	10	Cobalt–chromium	CAD/CAM Lost wax casting	Brass die	-	Profilometry	12	CAD/CAM	67.1 ± 15.67	100
								Lost wax casting	132.9 ± 27.9	
Chang, 2019 [40]	10	Cobalt–chromium	CAD/CAM Laser sintering Lost wax casting	Zirconia die	Maxillary molar	3D superimposition	-	CAD/CAM	89 to 178	107
								Laser sintering	110 to 120	
								Lost wax casting	63 to 84	
Dahl, 2018 [41]	18	Cobalt–chromium	CAD/CAM Lost wax casting Laser sintering	Acrylic tooth model	Maxillary incisor	3D superimposition	-	CAD/CAM	44 ± 49	52
								Laser sintering	63 ± 24	
								Lost wax casting	49 ± 32	
Mai, 2017 [42]	1	Cobalt–chromium	CAD/CAM	Metal die	Premolar	Impression replica 3D superimposition	Vertical hemisection	160 to 260		200
Tan, 2008 [44]	30	20 titanium 10 high noble metal alloy	CAD/CAM Lost wax followed by computer-aided milling Lost wax followed by conventional milling	Acrylic tooth models	Maxillary molar	Photogrammetry	4	CAD/CAM	79.43 ± 25.46	59
								Lost wax and computer aided milling	73.12 ± 24.15	
								Lost wax and conventional milling	23.91 ± 9.8	
Besimo, 1997 [45]	10	Titanium	CAD/CAM	Acrylic tooth models	14 crowns originally (4 incisors, 2 canines, 4 premolars, and 4 molars), with 4 cracked during milling	Scanning electron microscopy	109 to 205 circumferential measurements per crown at 100 µm intervals	21.2 to 81.6		51
Ferrari, 1994 [46]	6	Gold	Lost wax casting	Extracted human teeth	Maxillary molar	Scanning electron microscopy impression replica	-	Scanning electron microscopy	10 to 250 (mean = 130)	127
								Impression replica	10 to 240 (mean = 124)	
White, 1993 [47]	35	Base metal alloy	Lost wax casting	Extracted human teeth	Premolar	Direct view microscopy	12	19.7 to 50.8		35

**Table 2:** Critical appraisal outcomes.

	D1	D2	D3	D4	D5	D6	D7	D8
Berger, 2022 [38]	+	+	+	+	+	−	+	+
Chou, 2021 [43]	+	+	+	+	+	+	+	+
Gholamrezaei, 2020 [39]	+	+	+	+	+	unclear	+	+
Chang, 2019 [40]	+	+	+	+	−	−	+	+
Dahl, 2018 [41]	+	+	+	+	+	+	+	+
Mai, 2017 [42]	+	+	+	+	+	+	+	+
Tan, 2008 [44]	+	+	+	+	+	+	+	+
Besimo, 1997 [45]	+	+	+	+	+	−	+	+
Ferrari, 1994 [46]	+	+	+	+	+	unclear	+	+
White, 1993 [47]	+	+	+	+	+	+	+	+

D1: Were the criteria for inclusion in the sample clearly defined? D2: Were the study subjects and the setting described in detail? D3: Was the exposure measured in a valid and reliable way? D4: Were objective, standard criteria used for measurement of the condition? D5: Were confounding factors identified? D6: Were strategies to deal with confounding factors stated? D7: Were the outcomes measured in a valid and reliable way? D8: Was appropriate statistical analysis used?

**Table 3:** Comparison between lost wax casting and CAD/CAM for all-metal crown fabrication.

	Mean ± SD	t Stat (df)	*P* *
CAD/CAM	103.21 ± 58.56	1.057 (10)	0.315
Lost wax casting	71.60 ± 43.94		

* Significant < 0.05. SD, standard deviation; df, degree of freedom.

## Data Availability

The data that support the findings of this study are available from the corresponding author upon reasonable request.
